# Clinical and Biochemical Characteristics of COVID-19 in a Primary Care Center in the South Batinah Region of Oman

**DOI:** 10.7759/cureus.25110

**Published:** 2022-05-18

**Authors:** Shaikh M Naeem, Firdous Jahan, Moazzam Khan, Muhammad Siddiqui

**Affiliations:** 1 Department of Family Medicine, College of Medicine and Health Sciences, National University of Science and Technology, Rustaq, OMN; 2 Department of Family Medicine, College of Medicine and Health Sciences, National University of Science and Technology, Sohar, OMN; 3 Department of Research, Saskatchewan Health Authority, Regina, CAN

**Keywords:** risk factors, primary healthcare services, clinical characteristics, biochemical biomarker, covid-19

## Abstract

Objectives

The main purpose of this study was to identify the clinical and biochemical profile of coronavirus disease 2019 (COVID-19) and sex differences in Rustaq Polyclinic in South Batinah Governorate.

Methods

This study is a retrospective chart review of COVID-19 patients diagnosed in Rustaq Polyclinic in the South Batinah region of Oman. The medical charts of 150 patients from November 10, 2020, to November 24, 2021, at Rustaq Polyclinic were retrospectively reviewed, and clinical and laboratory parameters were extracted. Information regarding the patients’ demographics, risk factors, clinical symptoms or signs, and laboratory findings on admission were obtained. Statistical analysis was performed using the Statistical Package for Social Sciences (SPSS) software version 24.0 (IBM Corp., Armonk, NY, USA).

Results

A total of 150 study participant data were added to the analysis. Of those, 97 (64.7%) were male and 53 (35.3%) were female, with a median age of 32 and 39 among males and females, respectively. Male and female age (p=0.017) and marital status (p=0.003) were significantly different. No significant difference (p≥0.05) was observed between male and female study participants regarding their history of travel, history of contact with COVID-19-positive patients, diabetes, hypertension, stroke, and home management. Similarly, no significant difference (p≥0.05) was observed between males and females regarding their clinical presentations.

Conclusion

Many symptomatic patients have shown influenza-like symptoms such as fever, respiratory symptoms such as cough, sore throat, headache, myalgia, and loss of smell. The spectrum of symptomatic infection ranges from mild to severe, and most infections are not severe. It is imperative to equip ourselves with adequate understanding and protective measures such as vaccination and diverse treatment modalities against the constantly changing nature of novel coronaviruses.

## Introduction

Respiratory coronaviruses spread via direct contact with infected secretions, or both small and large aerosol droplets spread the infection [1]. Transmission occurs when respiratory droplets are generated via coughing, sneezing, talking, or direct contact with susceptible mucosal surfaces, such as the eyes, nose, or mouth. The common symptoms are fever, cough, dyspnea, and chest X-ray showing infiltrates [2]. Other common symptoms are aches and pains, sore throat, diarrhea, conjunctivitis, headache, loss of taste or smell, a rash on the skin, or discoloration of fingers or toes [3]. Severe symptoms are difficulty breathing or shortness of breath, chest pain or pressure, and speech or motor movement loss [4].

Although many reported infections are not severe, approximately 20% of confirmed patients have had a critical illness (including respiratory failure, septic shock, or other organ failure requiring intensive care) [1]. The fatality of the cases mainly occurs in patients having underlying medical comorbidities [1,5]. Around one in every five people infected with COVID-19 develops difficulty breathing and requires hospital care. People who are aged over 60 years and people who have underlying medical conditions such as diabetes, heart disease, respiratory disease, or hypertension are among those who are at greater risk. The WHO also advises that all confirmed cases, even mild cases, should be isolated in health facilities to prevent transmission and provide adequate care [6-8]. The two most common symptoms reported in the literature are cough and fever [1]. Disease severity and complications depend on comorbidities and immune system responses in populations [9-11]. Pneumonia is the most frequent manifestation and can progress to acute respiratory distress syndrome (ARDS).

Rapid techniques that can be used to detect coronaviruses from nasopharyngeal samples include reverse transcription-polymerase chain reaction (RT-PCR) and immunofluorescence antigen detection assays. The laboratory tests to assess severity and diagnosis are complete blood count, coagulation testing, liver and renal function tests, serum electrolytes, C-reactive protein, chest X-ray, and chest CT scan [12,13]. Suspected individuals with COVID-19 are advised to wear masks and keep a physical distance. Coronaviruses cause community-acquired upper respiratory tract infections, occurring sporadically or in outbreaks of variable size, and probably also play a role in severe respiratory infections [14]. The elderly population and people who have underlying medical conditions such as diabetes, heart disease, respiratory disease, or hypertension are among those who are at greater risk.

Understanding the clinical characteristics of COVID-19 helps in the early detection of the disease, appropriate management, and prevention of spread in the community. This study describes the demographic data, clinical characteristics, comorbidities, incubation periods, and outcomes of COVID-19-positive patients. The main purpose of this study was to identify the clinical and biochemical profile of COVID-19 and sex differences in Rustaq Polyclinic in South Batinah Governorate, Oman.

## Materials and methods

This study is a retrospective chart review of COVID-19 patients diagnosed in Rustaq Polyclinic in the South Batinah region of Oman. The medical charts of 150 patients from November 10, 2020, to November 24, 2021, at Rustaq Polyclinic were retrospectively reviewed, and clinical and laboratory parameters were extracted. A confirmed case of COVID-19 was defined as a positive result on a real-time reverse transcription-polymerase chain reaction (RT-PCR) assay of nasal and pharyngeal swab specimens. Only positive cases were included in the study.

The study was approved by the Institutional Ethical Review Committee, Oman. Data were collected without direct contact with patients, so informed consent was not required. All data were extracted from the patients’ electronic records. Information was gathered by a registered medical officer who was not involved in the assessment or treatment of the patients studied. Information regarding the patients’ demographics, risk factors, clinical symptoms or signs, and laboratory findings on admission were obtained.

Data analysis

Statistical analysis was performed using the Statistical Package for Social Sciences (SPSS) software version 24.0 (IBM Corp., Armonk, NY, USA). Categorical data were expressed in frequencies and percentages. Parametric continuous variables were represented as means with standard deviations, and nonparametric data were presented as medians with interquartile ranges. An independent sample t-test was used for parametric data, and the Mann-Whitney U test was utilized for nonparametric data to compare blood marker differences between genders. Chi-square tests were utilized to compare categorical variables between genders. Statistical tests were two-tailed with a significance level of p<0.05.

## Results

A total of 150 study participants’ data were added to the analysis. Of those, 97 (64.7%) were male and 53 (35.3%) were female, with a median year of age of 32 and 39 among males and females, respectively. A significant difference was observed in age (p=0.017) and marital status (p=0.003) between males and females (Table 1). No significant difference (p≥0.05) was observed between male and female study participants regarding their history of travel, history of contact with COVID-19-positive patients, diabetes, hypertension, stroke, and home management.

**Table 1:** Selected demographic and risk factors *Median (interquartile range) CAD: coronary artery disease n (%): number (percentage)

	Male (n (%))	Female (n (%))	Total (n (%))	p-value
Age*	32 (15)	39 (22)	34.5 (17.25)	0.017
Marital status				0.003
Married	59 (60.8)	45 (84.9)	104 (69.3)	
Single	38 (39.2)	8 (15.1)	46 (30.7)	
Travel history				1.0
No	96 (99)	53 (100)	149 (99.3)	
Yes	1 (1)	0	1 (0.7)	
History of contact				0.51
No	50 (51.5)	28 (52.8)	78 (52)	
Yes	47 (48.5)	25 (47.2)	72 (48)	
Attended large gathering				1.0
No	96 (99)	53 (100)	149 (99.3)	
Yes	1 (1)	0	1 (0.7)	
Treated at home				0.29
No	4 (4.1)	0	4 (2.7)	
Yes	93 (95.9)	53 (100)	146 (97.3)	
Refer for hospitalization				0.54
No	95 (97.9)	53 (100)	148 (98.7)	
Yes	2 (2.1)	0	2 (1.3)	
Diabetes				0.64
No	85 (87.6)	49 (92.5)	134 (89.3)	
Yes	5 (5.2)	2 (3.8)	7 (4.7)	
Hypertension				0.59
No	86 (88.7)	48 (90.6)	134 (8.3)	
Yes	6 (6.2)	4 (7.5)	10 (6.7)	
Stroke				1.0
No	96 (99)	53 (100)	149 (99.3)	
Yes	1 (1)	0	1 (0.7)	
Asthma				0.24
No	94 (96.9)	49 (92.5)	143 (95.3)	
Yes	3 (3.1)	4 (7.5)	7 (4.7)	
CAD				1.0
No	96 (99)	53 (100)	149 (99.3)	
Yes	1 (1)	0	1 (0.7)	
Smoking				1.0
No	96 (99)	53 (100)	149 (99.3)	
Unknown	1 (1)	0	(0.7)	

About two-thirds of the patients experienced fever (74%) without nasal discharge (78.7%). The majority of patients did not experience nausea or vomiting (96.7%), diarrhea (95.3%), fatigue (98.7%), and loss of smell (86.7%). No significant difference (p≥0.05) was observed between males and females regarding their clinical presentations (Table 2).

**Table 2:** Patients’ signs and symptoms at presentation n (%): number (percentage)

	Male (n (%))	Female (n (%))	Total (n (%))	p-value
Fever				0.89
No	24 (24.7)	15 (28.3)	39 (26)	
<3 days	58 (59.8)	30 (56.6)	88 (58.7)	
4-10 days	15 (15.5)	8 (15.1)	23 (15.3)	
Nasal discharge				0.27
No	73 (75.3)	45 (84.9)	118 (78.7)	
<3 days	17 (17.5)	7 (13.2)	24 (16)	
4-10 days	7 (7.2)	1 (1.9)	8 (5.3)	
Sore throat				0.69
No	64 (66)	32 (60.4)	96 (64)	
<3 days	27 (27.8)	16 (30.2)	43 (28.7)	
4-10 days	6 (6.2)	5 (9.4)	11 (7.3)	
Cough				0.49
No	53 (54.6)	25 (47.2)	78 (52)	
<3 days	35 (36.1)	20 (3.7)	55 (36.7)	
4-10 days	9 (9.3)	8 (15.1)	17 (11.3)	
Sputum production				1.0
No	96 (99)	53 (100)	149 (99.3)	
Yes	1 (1)	0	1 (0.7)	
Shortness of breath				0.54
No	95 (97.9)	53 (100)	148 (98.7)	
Yes	2 (2.1)	0	2 (1.3)	
Headache				0.45
No	69 (71.1)	41 (77.4)	110 (73.3)	
<3 days	22 (22.7)	11 (20.8)	33 (22)	
4-10 days	6 (6.2)	1 (1.9)	7 (4.7)	
Nausea or vomiting				0.63
No	94 (96.9)	51 (96.2)	145 (96.7)	
<3 days	2 (2.1)	2 (3.8)	4 (2.7)	
4-10 days	1 (1)	0	1 (0.7)	
Diarrhea				1.0
No	92 (94.8)	51 (96.2)	143 (95.3)	
<3 days	5 (5.2)	2 (3.8)	7 (4.7)	
Fatigue				0.12
No	96 (100)	51 (96.2)	147 (98.7)	
<3 days	0	2 (3.8)	2 (1.3)	
Myalgia or arthralgia				0.09
No	76 (78.4)	35 (67.3)	111 (74.5)	
<3 days	16 (16.5)	16 (30.8)	32 (21.5)	
4-10 days	5 (5.2)	1 (1.9)	6 (4)	
Loss smell				0.65
No	84 (86.6)	46 (86.8)	130 (86.7)	
<3 days	10 (10.3)	4 (7.5)	14 (9.3)	
4-10 days	3 (3.1)	3 (5.7)	6 (4)	

**Figure 1:**
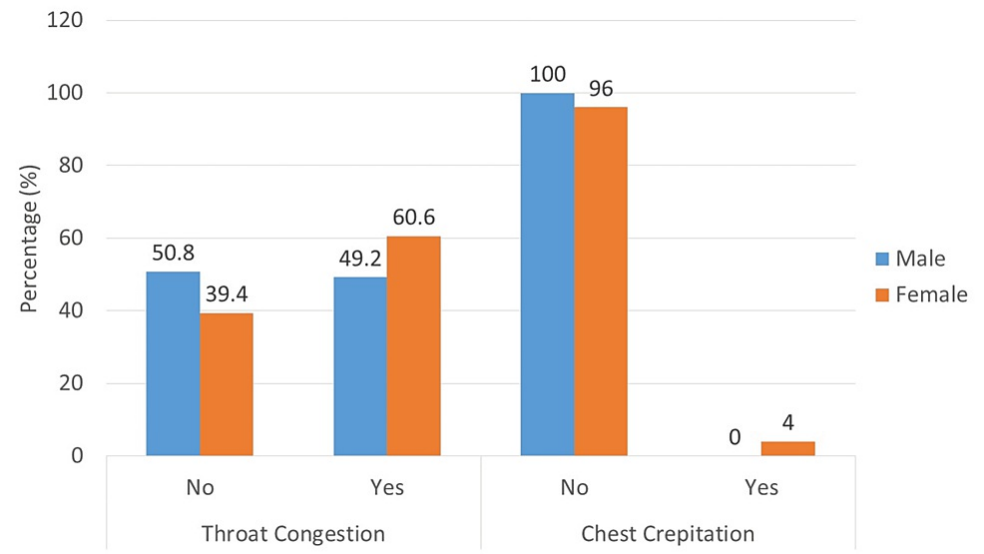
Throat congestion and chest crepitation

Nearly half (53%) of the study patients had throat congestion, and only 1.5% had chest crepitation on auscultation (Figure 1).

The routine blood test results and biochemical parameters are shown in Table 3. No difference was observed (p>0.05) in the blood markers between male and female study participants.

**Table 3:** Patients’ blood test results and biochemical parameters *Median (interquartile range) ^#^Mean±standard deviation SBP: systolic blood pressure, DBP: diastolic blood pressure

	Male	Female	Total	p-value
Temperature* (centigrade)	37 (1.7)	37 (0.6)	36.9 (1.7)	0.51
Respiratory rate* (breath/minute)	18 (4.5)	18 (1.5)	18 (4.5)	0.81
Oxygen saturation* (%)	99 (2.8)	99 (1.5)	98.5 (1.8)	0.68
SBP^# ^(mmHg)	122.1±12.9	128.5±15.6	128.2±16.5	0.13
DBP^# ^(mmHg)	74.6±10.1	76.3±11.4	80.7±2.9	0.61
Leucocytes* (1,000/uL)	4.3 (2.6)	4.5 (3.7)	4.3 (2.8)	0.50
Neutrophils* (1,000/uL)	56 (23)	52 (48)	54 (23.3)	0.81
Lymphocytes* (1,000/uL)	26.5 (15)	29 (37.4)	27.7 (18.8)	0.92
Platelets^# ^(1,000/uL)	169.8±57.9	249.8±68.5	195.3±70.9	0.01

## Discussion

Three common clusters of symptoms that have been identified in COVID-19 are as follows: cough, sputum, shortness of breath, and fever; musculoskeletal symptom cluster with muscle and joint pain, headache, and fatigue; and digestive symptom cluster with abdominal pain, vomiting, and diarrhea [15]. It takes 5-6 days from when someone is infected with the virus for symptoms to show; however, it can take up to 14 days for people with mild symptoms who are otherwise healthy to manage their symptoms at home [16]. In our retrospect data, the majority of the patients did not have any significant chest examination findings, and oxygen saturation was within the normal range. Literature has reported that clinical appearances of COVID-19 and radiology are variable [17].

Studies reported the prevalence of self-reported symptoms or clinician observed features in adults with confirmed coronavirus derived from nasopharyngeal swabs [18]. In this study, nearly half of the patients reported COVID-19 contact, and almost all of them were treated at home (Table 1). Fever was the consistent symptom in this study conducted retrospectively in one of the primary healthcare centers in Oman; about two-thirds of the patients experienced fever and dry cough. One-third of the patients had a headache, and some of them had myalgia and anosmia (Table 2). Grant et al. reported that fever and cough are the most prevalent symptoms of adults infected by SARS-CoV-2 [19]. However, a large number of populations did not have any symptoms.

In this study, nearly half of the patients had throat congestion, but no other significant examination finding was noticed (Figure 1). This study revealed significant leucopenia, neutropenia, and thrombocytopenia (Table 3). Alsofayan et al. reported from Saudi Arabia that a multicenter retrospective study revealed that fever and cough were the most common symptoms of COVID-19 [20]. However, asymptomatic carriers and healthcare workers play a critical role in disease transmission. In blood pictures, lymphocytopenia occurred in 37.5% of patients [19,20].

Clinical characteristics are variable in presentation; however, infectivity is higher immediately before and soon after the onset of symptoms. Infected patients can remain infectious for about two weeks. Ten days from the appearance of the initial symptoms, there is still the risk that a patient is still infectious. Clinical findings may not be present at the time of the clinical encounter. Literature reported that SARS-CoV-2 infection was associated with lower odds of dying, and 65 years or older individuals affected by obesity, diabetes, and asthma or those who are immunodepressed owing to cancer and other conditions are at a higher risk of hospitalization and of dying of COVID-19. Older age, sex, and underlying comorbidities were principal risk factors for illness severity at reinfection, and in particular, men have higher mortality and severe morbidity possibly explained by hormonal and/or genetic differences between sexes [21,22].

COVID-19-related illness can occur in otherwise healthy individuals of any age, but severe manifestations predominantly occur in adults with advanced age or specific underlying medical comorbidities.

Study limitations

This data was conducted retrospectively; some clinical features might have been missed and were not documented. The study sample was limited to one health center, possibly excluding a significant sample of other healthcare facilities in the area, so it cannot be generalized to the whole population. Therefore, more prospective study data is recommended at different primary care health centers.

## Conclusions

Coronavirus disease 2019 (COVID-19) is mainly an infectious disease of the respiratory system transmitted through air droplets, and pulmonary symptoms constitute the main presentations of this disease. The spectrum of symptomatic infection ranges from mild to critical; most infections are not severe. Many symptomatic patients have influenza-like symptoms such as fever and respiratory symptoms such as cough, sore throat, headache, myalgia, and loss of smell.
